# The School Doctor and the Tuberculous Child

**Published:** 1916-06-24

**Authors:** 


### THE SCHOOL DOCTOR.

### The School Doctor and the Tuberculous Child.

TUBERCULOSIS in public elementary schools is less common than hospital experience might lead one to suppose. Our own school statistics give a percentage varying from .8 per cent. to 3 per cent. of phthisis; and, while it is true that the ordinary noisy conditions of school medical inspection are singularly unfavourable to the early diagnosis of tubercle, special investigations undertaken by experts have confirmed these low figures. Professor Granchet and his colleagues, using the most refined methods of diagnosis, found only three children with active phthisis among 4,226 scholars of Paris. If, however, evidence of *tuberculous infection* is sought, and children with clinical signs of healed lung lesions, or of involvement of the bronchial glands, are included, a very large proportion of any school population must be classified as tuberculous. Granchet found 15 per cent. of his 4,226 children undoubtedly affected; later observers, especially those using $x$-rays or tuberculin tests, have recorded percentages as high as 80 per cent. All cases of malnutrition without obvious cause are now suspected of latent tubercle; in a special investigation conducted in London in 1913 Dr. Thomas found that 25 per cent. of cases of severe malnutrition were due to this cause. In much of the ill-health formerly attributed to "over-pressure" the tubercle bacillus is the real culprit.

### THE PROPAGATION OF TUBERCULOSIS IN SCHOOLS.

Very few authorities believe that the school plays any important part in the spread of tuberculosis, and the opinion of the International Tuberculosis Congress of 1905 that "tuberculosis in the child is nearly always contracted in the home" is now generally accepted as correct. The main force of the anti-tuberculosis campaign must therefore be directed through the family rather than the school, and a child once recognised as the subject of active tubercle becomes the charge of the tuberculosis officer rather than of the school doctor until the disease is definitely arrested. The obvious administrative arguments for this procedure are supplemented by the growing testimony of school medical officers (London and Sheffield Reports, 1914) that palliative measures such as day open-air schools give thoroughly disappointing results with these children. They need whole-hearted and persistent treatment in an institution which is primarily a sanatorium and only incidentally a school.

### AN ADMINISTRATIVE QUESTION.

There is another reason for making "a clear distinction between the diagnosis of the presence of tubercular tissue and the diagnosis of active tuberculosis." The great mass of children with "latent" or "glando-pulmonary" lesions tend to a spontaneous cure. To invoke the machinery of a tuberculosis department to deal with them is as extravagant as it is confusing to the public, who are being laboriously trained to regard tuberculosis as a dangerous disease requiring energetic and immediate treatment. Experience has shown that few things damage the reputation of the school doctor or cause friction so much as over-enthusiasm in the matter of notification. This does not imply a policy of inaction. The school doctor has rightly been called "the first line of defence" against tuberculosis, and he finds ample scope for his energies in watching these children's progress and meanwhile in strengthening their resistance. Re-inspection at frequent intervals of all suspects is now the usual school routine; where possible, temperature records or fortnightly weighings are made. This is, indeed, almost the only useful purpose the weighing-machine can serve in the elementary school.

### FRESH AIR AND GOOD FOOD.

The phenomenal success of day open-air schools in Central Europe and America has led to dangerous misconceptions as to the part that fresh air plays in increasing the individual's resistance to tubercle. As Dr. Kerr has pointed out children do not live on air, and fresh air acts mainly not by nourishing but by increasing the desire for nourishment and the capacity for assimilating it. The striking results of the parent of open-air schools, the Forest School of Charlottenburg, were largely due to the provision of three good meals a day and two hours of midday rest, its imitators elsewhere have only succeeded in so far as they have copied the routine in its entirety. "Playground classes," which keep the children out of doors a certain number of hours daily, but provide no extra nourishment or sleep or warm wraps, are satisfactory enough for normal children, but almost useless for the tuberculous, and may do actual harm to the delicate, underfed child.

Where one is dealing with a ubiquitous infective agent like the tubercle bacillus, and a generally susceptible population, rational preventive measures must embrace the whole people without exception. This is certainly true of children. That *all* schools should be in a great measure open-air schools, that *all* scholars should spend more of their time in the playground, and that adequate nourishment should be within reach of *every* child, are now accepted maxims of school hygiene. Not until they have been put into practice can the school be said to be taking its full share in the elimination of tuberculosis.

